# A new paradigm for tumor immune escape: β-catenin-driven immune exclusion

**DOI:** 10.1186/s40425-015-0089-6

**Published:** 2015-09-15

**Authors:** Stefani Spranger, Thomas F. Gajewski

**Affiliations:** University of Chicago, 5841 S. Maryland Ave., MC2115, Chicago, IL 60637 USA; University of Chicago, 929 E 57th Street, GCIS, Chicago, IL 60637 USA

**Keywords:** Tumor microenvironment, Immune evasion, Checkpoint inhibition, T-cell infiltration

## Abstract

Increasing evidence is emerging that immunotherapeutic interventions, including checkpoint blockade, are predominantly effective in patients with a pre-existing T cell-inflamed tumor microenvironment. Understanding the mechanisms leading to a non-T cell-inflamed microenvironment are crucial for the development of novel treatment modalities to expand the fraction of patients benefiting from immunotherapy. Based on the hypothesis that one source of inter-patient heterogeneity would lie at differential activation of specific oncogene pathways within the tumor cells themselves, our group recently observed that tumor-cell intrinsic activation of the WNT/β-catenin pathway correlates with absence of T cells from the microenvironment in metastatic melanoma. Genetically-engineered mouse models confirmed a causal relationship, via a mechanism of failed Batf3-lineage dendritic cell recruitment. Hence, tumor cell-intrinsic activation of β-catenin is the first oncogenic pathway demonstrated to exclude the anti-tumor immune response, revealing a potential therapeutic target for improving immunotherapy responsiveness.

## Background

Despite the growing success of immunotherapy in the treatment of advanced cancer, it is clear that only a subset of cancer patients experience clinical benefit from these interventions. Recent biomarker observations have supported the premise that most clinical responders to anti-PD-1 mAb, anti-CTLA-4 mAb, and cancer vaccines show a pre-existing T cell-inflamed tumor microenvironment at baseline [1–3]. Post-treatment biopsies from melanoma patients receiving anti-PD-1 have revealed an expanded number of proliferating CD8^+^ T cells penetrating deep within the tumor microenvironment [4]. These data are consistent with preclinical data demonstrating that most of the therapeutic effect of checkpoint blockade can be attributed to re-activation of CD8^+^ T cells already present within the tumor [5]. Patients with tumors that completely lack adaptive immune cell infiltration may require novel therapeutic interventions to restore T cell entry and enable responsiveness to our current immunotherapies. As such, understanding the underlying mechanisms of T cell exclusion has become a critically important biologic question with clinical relevance.

## Main text

Our laboratory has been pursuing three potential levels of inter-patient heterogeneity that could explain the presence or absence of the T cell-inflamed tumor microenvironment phenotype in individual patients: somatic differences at the level of the tumor cells, germline polymorphism differences at the level of the host, and environmental differences at the level of the intestinal microbiota. Beginning with the hypothesis that activation of specific oncogene pathways might mediate immune exclusion in tumors from individual patients [6], we utilized metastatic melanoma data from 266 tumor samples from The Cancer Genome Atlas (TCGA) and segregated them based on the presence or absence of a gene signature indicative of the T cell-inflamed phenotype. Using these same tumors, exome sequencing and pathway analysis were performed, which revealed that 48 % of the non-T cell-inflamed tumors showed evidence for activation of the Wnt/β-catenin pathway. In order to determine whether activation of the β-catenin pathway was causally related to immune exclusion, autochthonous mouse models were developed utilizing a melanocyte-specific, tamoxifen-regulated Cre [7], paired with conditional Braf^V600E^ induction, PTEN deletion, and/or β-catenin stabilization [8, 9]. While a T cell infiltrate was indeed observed in tumors driven by Braf^V600E^ and PTEN deletion, this T cell infiltrate was completely absent in tumors that additionally expressed active β-catenin. To further investigate the mechanism of T cell exclusion, an SIY antigen-reporter mouse (Rosa26-Lox-Stop-Lox-SIY) [10] was used in combination with adoptive transfer of SIY-specific TCR-transgenic T cells (2C T cells). Although brisk activation and tumor accumulation of the transferred T cells was observed in in Braf^V600E^/PTEN mice, no such activation or accumulation was observed in mice bearing tumors additionally expressing active β-catenin. This observation prompted analysis of the antigen-presenting cell compartment in both tumor types, which revealed a significant reduction of CD103/CD8α dendritic cells (DCs) in β-catenin-expressing tumors. Rescue experiments utilizing intratumoral injection of Flt3 ligand-derived DCs showed restoration of T cell infiltration. To probe more deeply into the mechanism of failed recruitment of Batf3-lineage DCs, gene expression profiling of the two tumor genotypes was performed with a focus on chemokines. These studies revealed Braf^V600E^/PTEN tumor cells were capable of secreting the chemokine CCL4, whereas no CCL4 expression was observed with tumor cells that additionally expressed stabilized β-catenin. These findings were confirmed using tumor cell lines derived from both mouse models as well as with human melanoma cell lines that contained or lacked active β-catenin signaling. The ability of active β-catenin to prevent CCL4 gene expression was mapped to induction of a transcriptional repressor ATF3, which ChIP assays confirmed was binding the CCL4 promoter. To explore in vivo efficacy of checkpoint blockade with these two tumor genotypes, mice were treated with a combination of anti-CTLA4 and anti-PD-L1 mAbs. Although this treatment delayed tumor outgrowth in Braf^V600E^/PTEN mice, no therapeutic effect was observed in mice bearing tumors that additionally expressed active β-catenin. Responsiveness to checkpoint blockade was restored through direct injection of FLt3L-derived DCs, demonstrating the rate-limiting role of proper DCs for activating tumor antigen-specific T cells, which in turn allowed for response to checkpoint inhibition.

## Conclusion

The observation that oncogenic pathways within tumor cells have the capability to directly impact the anti-tumor immune response is likely to have impact on both the research directions in the field and also on prioritization of clinical development of new targeted inhibitors. Evasion from the immune system is a well-known phenomenon, but thus far it has been focused on immune-mediated selection for antigen-loss variants, combined with upregulation of immune inhibitory mechanisms that thwart the efforts of remaining T cells having intermediate affinity TCRs for remaining antigens. However, these mechanisms failed to explain the existence of the non-T cell-inflamed tumor microenvironment phenotype, which contain tumor cells that express antigens but nonetheless fail to support a dialogue with the host immune response. The non-T cell-inflamed phenotype, in fact, is the most common pattern observed in human samples and in TCGA data analysis across a spectrum of tumor types, and so understanding the biology of this mechanism of immune resistance is paramount. It is likely that activation of the Wnt/β-catenin pathway is relevant for immune evasion in additional cancers beyond melanoma. Preliminary data have indicated that the β-catenin pathway is associated with T cell exclusion in bladder cancer and also in head and neck cancer [11]. Inasmuch as β-catenin activation accounted for 48 % of non-T cell-inflamed melanomas, other oncogene pathways likely contribute to immune exclusion in the remainder of these tumors, and similarly in other cancers as well. Activation of the Ras/Raf pathway has already been investigated to some degree in human patients and early studies have suggested an increased infiltration by T cells after administration of Braf inhibitors [12]. However, it is not yet clear if these T cells are tumor-specific or whether their recruitment leads to productive T cell activation versus dysfunction. The PI3K/PTEN pathway is also being investigated, but with somewhat contradictory data thus far. Analysis of samples from triple negative breast cancer patients has indicated that loss of PTEN is associated with presence of T cells within the microenvironment. In contrast, data presented on malignant melanoma has indicated the opposite [13, 14]. Therefore, cancer-type specific mechanistic studies might be needed to answer this question definitively. Continued interrogation of these and other oncogene pathways in the full range of tumor types should be stablished as a high research priority. As each candidate oncogene-driven mechanism of immune exclusion is validated mechanistically, then pharmacologic approaches to block these pathways should be integrated into combination studies in concert with immunotherapeutics such as anti-PD-1. It is tempting to speculate that a bidirectional iterative translational research program identifying molecular mechanisms of resistance immunotherapies and combination therapies will ultimately lead to an expansion of clinical impact to encompass the majority of cancer patients.

## Abbreviations

mAb: monoclonal antibodies
PD-1: Programmed death-1
CTLA-4: Cytotoxic T-lymphocyte-associated protein 4
DC: Dendritic cell
TCR: T cell receptor
TCGA: The Cancer Genome Atlas
CCL4: C-C-motive ligand 4
ChIP: Chromatin immunoprecipitation
ATF3: Activating transcription factor 3
